# Assessment of dentofacial growth deviation in juvenile idiopathic arthritis: Reliability and validity of three-dimensional morphometric measures

**DOI:** 10.1371/journal.pone.0194177

**Published:** 2018-03-13

**Authors:** Peter Stoustrup, Christian Kerting Iversen, Kasper Dahl Kristensen, Cory M. Resnick, Carlalberta Verna, Sven Erik Nørholt, Shelly Abramowicz, Annelise Küseler, Paolo Maria Cattaneo, Troels Herlin, Thomas Klit Pedersen

**Affiliations:** 1 Section of Orthodontics, Aarhus University, Aarhus, Denmark; 2 Section of Orthodontics, University of Bergen, Bergen, Norway; 3 Harvard School of Dental Medicine, Boston, MA, United States of America; 4 Department of Plastic and Oral Surgery, Boston Children's Hospital, Boston, MA, United States of America; 5 Clinic for Orthodontics and Pediatric Dentistry, University Center for Dental Medicine, University of Basel, Basel, Switzerland; 6 Department of Oral and Maxillofacial Surgery, Aarhus University Hospital, Aarhus, Denmark; 7 Section of Oral Maxillofacial Surgery, Aarhus University, Aarhus, Denmark; 8 Oral and Maxillofacial Surgery and Pediatrics, Emory University, Children’s Healthcare of Atlanta, Atlanta, GA, United States of America; 9 Department of Pediatrics, Aarhus University Hospital, Aarhus, Denmark; Medical University of South Carolina, UNITED STATES

## Abstract

**Introduction:**

Patients with juvenile idiopathic arthritis (JIA) and involvement of the temporomandibular joint (TMJ) often experience abnormal facial growth. Three-dimensional (3D) assessment of dentofacial growth deviation has become more common with advancement and commercialization of imaging technologies. However, no standardized guidelines exist for interpretation of 3D imaging in patients with JIA. The aim of this study was to propose and validate morphometric measures for the 3D radiographic assessment of dentofacial growth deviation in patients with JIA to enhance: 1) Description of dentofacial growth deviation; 2) Treatment planning; 3) Longitudinal follow-up.

**Methods:**

The study was conducted in a standardized sequential-phased approach involving: 1) Preliminary decision-making; 2) Item generation; 3) Test of content-validity; 4) Test of reliability; 5) Test of construct validity; 6) Establishment of final recommendations.

**Results:**

Twenty-one morphometric measures were evaluated. Based on results of reliability and validity-testing including subjects with JIA (n = 70) and non-JIA controls (n = 19), seven measures received a “high recommendation” score. Those measures were associated with posterior mandibular height, occlusal cant, mandibular asymmetry, mandibular inclination, and anterior/posterior lower face height. Nine other measures were “moderately recommended” and five received a “somewhat recommendation” score.

**Conclusion:**

Seven morphometric measures were considered very useful in the 3D assessment of growth deviation in patients with TMJ disease associated with JIA. These variables can be used to standardize the description of dentofacial deformities and to plan corrective interventions.

## Introduction

Juvenile idiopathic arthritis (JIA) is the most common rheumatic disease that occurs in children and adolescents [1]. Involvement of the temporomandibular joint (TMJ) occurs in up to 75–92% of patients with JIA [2, 3]. In growing individuals, arthritis of the TMJ may affect the intra-articular mandibular growth site situated in the condylar cartilage, leading to dentofacial deformity [4–6]. The resulting deformity is dependent on the duration, timing, extent and distribution (unilateral or bilateral) of arthritic involvement [7].

Dentofacial growth deviation in JIA with TMJ involvement has been well-described using two-dimensional (2D) cephalometric analyses [6]. However, three-dimensional (3D) imaging provides superior visualization of dentofacial structures compared to 2D techniques [8, 9]. With the introduction of cone-beam computed tomography (CBCT) to odontology, 3D imaging techniques have gained popularity in the assessment of dentofacial growth deviation over the past two decades[8, 9]. Several protocols for 3D radiographic evaluation of facial asymmetry and malocclusion have been published recently for non-JIA subjects [10–14]. In patients with JIA and TMJ involvement, 3D morphometric measures have been used to assess dentofacial abnormalities [15–17], but no standardized guidelines exist for the 3D radiographic evaluation of dentofacial growth deviation in these patients.

Most 3D morphometric research has focused on landmark identification and measurement accuracy [12]. Comparatively little attention has been devoted to the diagnostic efficacy of 3D imaging in patients with dentofacial deformities [9]. The morphologic characteristics of dentofacial deformities vary by etiology; arthritis-related deformities may differ from other groups (e.g. hemifacial microsomia, post-traumatic). Extrapolated evidence from the literature suggests that the content of a 3D analysis should be dictated by: 1) the characteristics of the deformity within the patient group of interest, 2) the objectives of the 3D analysis (e.g. diagnosis, treatment planning, therapeutic efficacy), and 3) the sensitivity of analysis to reveal changes in the degree of deformity over time.

The aim of this project is to propose and validate morphometric measures for the 3D radiographic assessment of dentofacial growth deviation in JIA within the following three domains: 1) description of the deformity, 2) treatment planning, and 3) long-term assessment.

## Material and methods

Using a sequential-phased approach [18], this study included the following steps: 1) preliminary decisions, 2) item generation, 3) test of content-validity, 4) test of reliability, 5) test of construct validity, and 6) adjustments and establishment of final recommendations.

### Preliminary decisions

A working group comprised of four authors (PBS, CKI, PMC and TKP) was convened. The working group defined the study objective to propose evidence-based morphometric outcome measures for 3D radiographic orofacial evaluation of patients with JIA in the following domains: 1) description of dentofacial deformity, 2) treatment planning, and 3) long-term assessment.

### Item generation

The working group identified 21 unique dentofacial morphometric measures based on traditional 2D cephalometric standards [8–15]. Anatomic landmarks and reference planes were defined based on current literature [6, 16, 19–26]. These measures were defined with the aim to describe dentofacial symmetry and skeletal relationships in sagittal, vertical and transverse dimensions. Four types of measures were defined: 1) inter-side differences in linear distances or angles, 2) angles at the intersection of predefined reference planes, 3) anterior/posterior facial height ratios, and 4) miscellaneous measures.

To assess inter-side differences in linear distances or angles (morphometric measures No 1–11), we defined the “asymmetry side” as the side with the smallest total posterior mandibular height (No.1), and measurements for the “asymmetry-side” were subtracted from the contralateral (larger) side. Subsequently, the “asymmetry side” was used for the calculation of the additional morphometric measures (No.2-11), depicting inter-side differences in the following way: “asymmetry-side” distance/angle subtracted from the distance/angle of the contralateral side. Subsequent measures (No. 2–11) could therefore demonstrate positive inter-side differences because the “asymmetry side” had been defined by the smallest total posterior mandibular height (No.1).

### Content-validity

Content-validity is defined as the level to which the content of the proposed measures reflects the underlying construct of the assessment. Six external experts, identified based on clinical expertise and research activity in this field, were invited to assess the content-validity of the measures defined in item generation. The external experts included three orthodontists (AK, CV, KDK) and three maxillofacial surgeons (SA, CMR, SEN). Content-validity testing was then performed by questionnaire, with assessment of each measure assessed by a Likert scale from 1–5 (1 = strongly disagree, 5 = strongly agree) in the three domains: 1) descriptive, 2) treatment planning, and 3) longitudinal assessment. An average score of 3.5 or above was considered a “high” score. For each measure, a unique statement was proposed for each domain:

Descriptive domain. Statement: This is an important morphometric measure for the overall description of the severity of dentofacial growth deviation in JIA.

Treatment planning domain. Statement: This is a useful morphometric measure in the planning of orthodontic/surgical management.

Longitudinal assessment domain. Statement: This is an important measure for the description of changes over time for dentofacial growth deviation in JIA (e.g. pre-/posttreatment or with growth).

In addition, the external experts were asked to assess the overall “strength of recommendation” (SOR) for each measure using a 100 mm visual analogue scale (0 mm = not important, 100 mm = extremely important). Finally, the experts were asked to rank the measures based on “overall importance” from 1 to 21 (1 = most important, 21 = least important).

#### Subjects

To test content validity of the measures defined in item generation, the study group included patients with JIA. These patients represent the Aarhus TMJ arthritis cohort, which is a population-based group that contains standardized longitudinal observational data about patients with JIA. The included 70 patients are consecutive patients referred to the Section of Orthodontics, Aarhus University, Denmark for a CBCT from February 2011 to April 2014. Data was retrieved retrospectively. Inclusion criteria were: 1) a diagnosis of JIA according to ILAR criteria [27, 28], and 2) a high quality full-face CBCT. Patients with a history of facial trauma or a congenital craniofacial anomaly were excluded. CBCTs obtained for routine orthodontic management from an age-matched non-JIA control group with no history of temporomandibular joint dysfunction were also included. Approvals from the Danish Health and Medicine authorities (3-3013-641/1) and the Danish Data Protection Agency (1-16-02-458-14) were obtained prior to the initiation of the study.

The retrospective retrieved CBCT examinations had been conducted in accordance with the manufacture instructions and in agreement with regulations approved by the Danish Health and Medicines authorities. A NewTom 5G, 18x16 cm field of view was used for all CBCTs. The image acquisition parameters included a scanning time of approximately 18 seconds, active radiation 3.6 sec (pulsed mode) with settings of 110 kV and 3–7 mA. All CBCT scans were constructed with a 0.30 mm isotropic voxel dimension. 3D data evaluation was conducted using Mimics software (Mimics® 18.0; Materialize Interactive Medical Image Control System, Leuven, Belgium). Anatomic landmarks were identified in all three planes of space (axial, sagittal, coronal). All proposed morphometric measurements were made for each subject by one author (CKI). Additionally, the radiographic appearance of each TMJ was categorized based on published criteria [17]:

1. Normal: Normal condylar shape with smooth and intact cortical outline/surface.
2. Abnormal: Condylar flattening or other changes in shape with smooth and intact outline and/or disruption of condylar outline with uneven surface due to cyst or erosion.

Each JIA subject was then assigned to one of the following groups: 1) JIA 1 (normal TMJs), 2) JIA 2 (unilateral abnormal TMJ), or 3) JIA 3 (bilateral abnormal TMJs).

### Test of reliability

To assess inter-rater variability, the same author repeated measurements for 30 subjects (selected using block randomization) a minimum of two weeks after the first assessment. These data were then used to calculate the smallest detectable difference for each measurement, defined as the smallest change that can be reliably observed between two consecutive observations (error of the measurement) [29, 30]. To evaluate inter-rater agreement, a second author (PBS) evaluated the same 30 subjects.

### Test of construct validity

Construct validity is defined as the degree to which the proposed outcome variables measure what they are intended to. To assess this, inter-group differences were calculated for each measurement. Associations between the inter-group results were then related to the following predefined hypotheses (H):

H1: Morphometric measures demonstrate greater inter-side vertical and sagittal asymmetry in JIA subjects compared to controls.
H2: JIA 2 and JIA 3 subjects demonstrate greater inter-side vertical and sagittal asymmetry compared to JIA 1 subjects.
H3: Occlusal plane canting and occlusal plane inclination are more pronounced in JIA 2 and JIA 3 subjects compared to JIA 1 subjects and controls.
H4: Mandibular retrognathia is more pronounced in JIA 3 subjects compared to the other groups.

### Establishment of final recommendations

A consensus-driven approach among all authors was used to establish final recommendations. Each measure was assigned a grade for each domain: highly recommended (+++), moderately recommended (++), somewhat recommended (+), not recommended (-). Because each domain was assessed separately, measures could receive higher recommendations for some domains than others.

#### Statistical analysis

Descriptive statistics were computed. Intra-class correlations coefficients (ICC) were calculated using a two-way random effect model to assess intra-rater and inter-rater correlation. An ICC > 0.70 was considered acceptable. Bland-Altman plots and the limits of agreement were used to calculate smallest detectable differences [31]. Construct validity was tested against the predefined hypotheses using analysis of variance (ANOVA). For measures with a significant ANOVA result, independent Student’s *t*-tests were then applied. The level of significance was set at p < 0.05.

## Results

### Preliminary decisions and item generation

**Fig 1:**
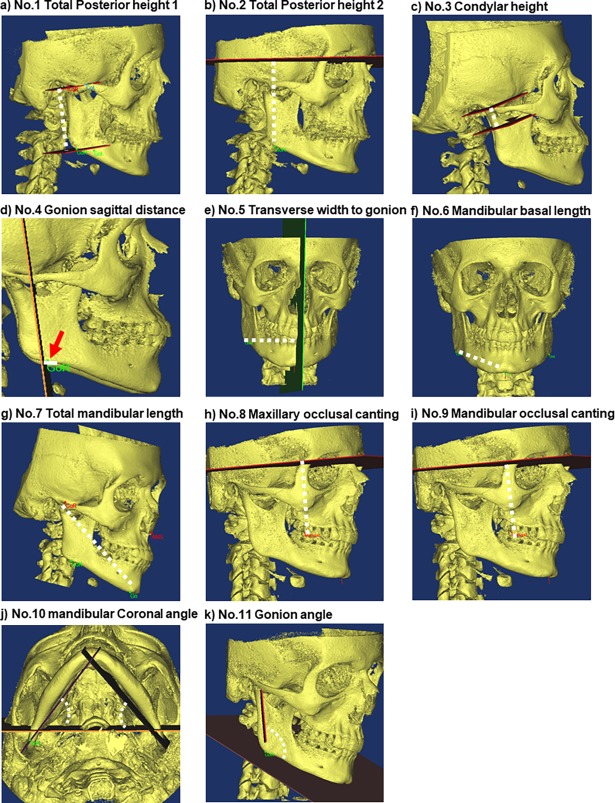
Morphometric measures No1-11. Outcomes are calculated as the inter-side differences in bilateral linear distances (mm) or bilateral angles (degrees). a) No.1, Total posterior mandibular height 1, mandibular level: Distance from ramus axial plane 1 –ramus axial plane 3 through Co-Go. b) No.2, Total posterior mandibular height 2 cranial level; distance from axial plane to Go. c) No.3, Condylar height; distance from Co to Ramus axial plane 2. d) No.4, Gonion sagittal distance; distance from coronal plane to Go. e) No.5 Transverse width to gonion:; distance from Sagittal plane to Go. f) No.6, Mandibular basal length; distance from Go to Gnathion (Gn). g) No.7, Total mandibular length; distance from Co to Pogonion (Pg). h) No.8, Maxillary occlusal canting; Distance from Axial plane to upper molar cusp (MolSup). i) No.9, Mandibular occlusal canting; Distance from Axial plane to the lower molar cusp (MolInf. j) No.10, Mandibular coronal angle (y-axis asymmetry); angle between Mandibular construction plane and the Coronal plane, k) No.11 Gonion angle; angle between Ramus Coronal plane and Mandibular Axial plane.

**Fig 2:**
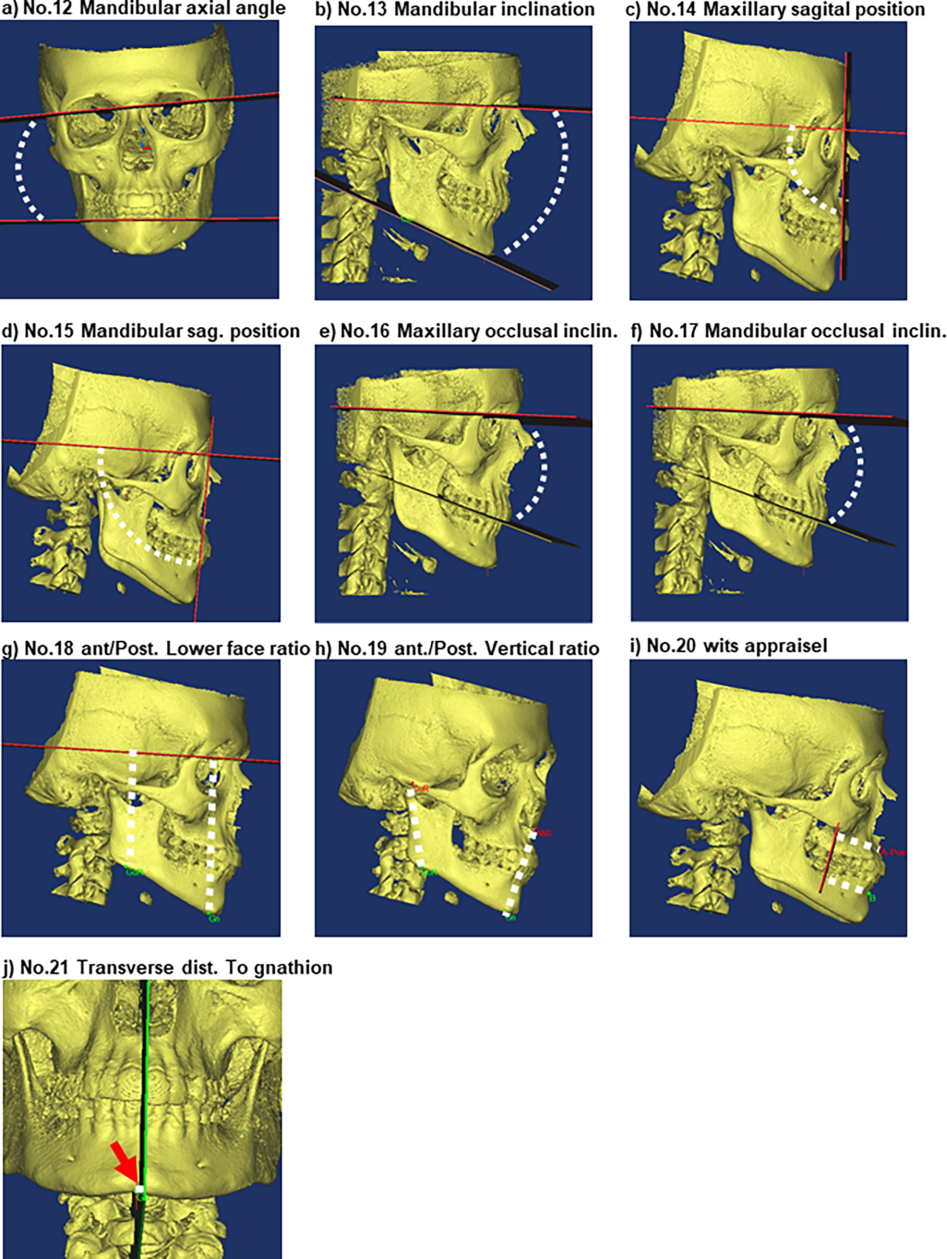
Morphometric measures No. 12–21. Outcomes in morphometric measures No.12–17 are presented as the angle between two predefined planes. No. 18–19 are defined as anterior/posterior height ratios. a) No.12, Mandibular axial angle (z-axis asymmetry); angle between Gonion axial Construction Plane and Axial plane. b) No. 13, Mandibular inclination; angle between Mandibular Axial plane and Axial plane. c) No.14, Maxillary sagittal position; angle between Axial plane and Maxillary protrusion plane. d) No.15, Mandibular sagittal position; angle between Axial plane and Mandibular protrusion plane. e) No.16, Maxillary occlusal inclination; angle between Axial plane and maxillary occlusal plane. f) No.17, Mandibular occlusal inclination; angle between Axial plane and mandibular occlusal plane. g) No.18, Posterior/anterior lower face height ratio (cranial level); distance from Axial plane to Go (average of right and left side) divided by distance from Axial plane to Gnathion. h) No.19, Posterior/anterior vertical ratio (mandibular level); distance from Condylion to Gonion (average of right and left side) divided by distance Anterior nasal spine to Gnathion. i) No.20, Wits appraisal; distance between A-point and molar Coronal construction Plane subtracted the distance from the B-point to molar Coronal construction Plane. j) No.21, Transverse distance from gnathion to midsagittal plane; distance between Gnathion perpendicular to Sagittal plane.

23 landmarks, 12 internal reference planes, and six side-specific reference planes were defined (Table 1, Table 2). From these, 21 morphometric measures were chosen for further evaluation: 1) Nine linear inter-side measurements (Fig 1, No. 1–9); 2) two angular inter-side measurements (Fig 1, No. 10–11); 3) six angles defined by reference planes (Fig 2, No. 12–17); 4) two anterior/posterior face height ratios (Fig 2, No. 18–19); and 5) two miscellaneous measures: Wits appraisal, defined as the distance between A-point and B-point measured along the occlusal plane, and the transverse distance from gnathion to the midsagittal reference-plane (Fig 2, No. 20–21).

**Table 1:** Definitions of landmarks and planes.

Anatomical landmark	Definition	Abbreviation
Sella	The centre of the hypophyseal fossa	**S**
Nasion	Midpoint between maxillary-nasal-frontal right and left junction chin	N
Basion	The most anterior-inferior point on the margin of the foramen magnum	Ba
Pogonion	The most anterior point of the mandible in relation to the coronal plane in the mandibular midline	Pg
A point	Deepest concavity on the anterior part of the maxilla at sagittal plane level in relation to the coronal plane	A
Gnathion	The most anterior/inferior border of the chin in the mandibular midline	Gn
B point	Deepest concavity on the anterior part of the mandible in relation to the coronal plane in the mandibular midline	B
Anterior nasal spine	The most anterior point of the anterior maxillary spine	ANS
Condylion, R	Most superior point and the midpoint of the mediolateral axis of the *right* condyle	CoR
Condylion, L	Most superior point and the midpoint of the mediolateral axis of the *left* condyle	CoL
Incisura, R	Lowest point in the concavity between processus coronoideus and processus condylaris- right	IncR
Incisura, L	Lowest point in the concavity between processus coronoideus and processus condylaris- left	IncL
Gonion, R	Most posterior/inferior point on the right mandibular ramus	GoR
Gonion, L	Most posterior/inferior point on the left mandibular ramus	GoL
Latero-Orbital point, R	Zygomatico-frontal suture at the medial aspect of the orbital wall, right	LO_R
Latero-Orbital point, L	Zygomatico-frontal suture at the medial aspect of the orbital wall left	LO_L
Midpoint upper incisors	Midpoint(incisal edge) or contact point between superior incisors	InS
Midpoint lower incisors	Midpoint(incisal edge) or contact point between inferior incisors,	InInf.
Midpoint between incisors	Vertical midpoint between upper and lower incisors	InS-InInf.
Cusp upper molar, R	Disto-Facial cusp 1 upper molar, right	MolSupR
Cusp upper molar, L	Disto-Facial cusp 1 upper molar, left	MolSupL
Cusp lower molar, R	Disto-Facial 1 lower molar, right	MolInfR
Cusp lower molar, L	Disto-Facial cusp 1 lower molar, left	MolInfL
**Plane**	**Definition**	**Abbreviation**
1.	S-Lo_R-Lo_L	Axial plane
2.	S-N- perpendicular to Axial plane	Sagittal plane
3.	S-perpendicular to the Sagittal plane perpendicular to the Axial plane	Coronal plane
4.	Gor-Gol-Gn	Mandibular axial plane
5.	MolSupR- MolSupL–InS-InInf.	Combined occlusal plane
6.	MolSupR-MolSupL-InS	Maxillary occlusal plane
7.	MolInfR- MolInfL-InInf	Mandibular occlusal plane
8.	MolSupR perpendicular to Combined occlusal plane and Sagittal plane	Molar coronal Construction Plane
9.	Gor-GoL-perpendicular to the Coronal plane	Gonion Axial construction plane
10.	GoR-GoL- perpendicular to the Axial plane	Gonion Coronal construction plane
11.	N–Pogonion—perpendicular. to Sagittal plane	Mandibular protrusion plane
12.	N–A-point–perpendicular.- to Sagittal plane	Maxillary protrusion plane:
**Side-specific planes (one in each side)**	**Definition**	**Abbreviation**
1.	Go-Gn-perpendicular to the Axial plane	Mandibular Construction plane
2.	Co-Go-Gn	Ramus sagittal plane
3.	Co-Go-perpendicular to the Ramus sagittal plane	Ramus coronal plane
4.	Through Co—perpendicular to Ramus sagittal plane -perpendicular to Ramus Coronal plane	Ramus Axial plane 1
5.	Through Inc- perpendicular to Ramus sagittal plane -perpendicular to Ramus Coronal plane	Ramus Axial plane 2
6.	Through Go- Perpendicular to Ramus sagittal plane -perpendicular to Ramus Coronal plane	Ramus Axial plane 3

**Table 2:** Definition of morphometric measures.

Reference Number of morphometric measure (No.)	Morphometric measures	Definition
	**Inter-side difference in bilateral linear distances**	**(Interside difference: largest side subtracted the “asymmetry side” No 1–11)**
1.	Total posterior mandibular height 1, (mandibular level)	Distance from ramus axial plane 1 –ramus axial plane 3 through Co-Go
2.	Total posterior mandibular height 2, (Cranial level)	Distance from Axial plane to Go
3.	Condylar height	Distance between Co and Ramus axial plane 2
4.	Gonion sagittal distance	Distance from Coronal plane to Go
5.	Transverse width to gonion	Distance from Sagittal plane to Go
6.	Mandibular basal length	Go-Gn
7.	Total mandibular length	Co-Pg
8.	Maxillary occlusal canting	Distance from Axial plane to MolSup
9.	Mandibular occlusal canting	Distance from Axial plane to MolInf
	**Inter-side difference in bilateral angles**	
10.	Mandibular coronal angle(y-axis asymmetry)	Angle between Mandibular construction plane and the Coronal plane (Inter-side difference)
11.	Gonion angle	Angle between Ramus Coronal plane and Mandibular Axial plane (Inter-side difference)
	**Angles between predefined planes**	
12.	Mandibular axial angle (z-axis asymmetry)	Angle between Gonion axial Construction Plane and Axial plane
13.	Mandibular inclination	Angle between Mandibular Axial plane and Axial plane
14.	Maxillary sagittal position	Angle between Axial plane and Maxillary protrusion plane
15.	Mandibular sagittal position	Angle between Axial plane and Mandibular protrusion plane
16.	Maxillary occlusal plane inclination	Angle between Axial plane and Maxillary occlusal plane
17.	Mandibular occlusal plane inclination	Angle between Axial plane and Mandibular occlusal plane
	**Anterior/posterior face height ratios**	
18.	Posterior/anterior lower face height ratio (Cranial level)	Distance from Axial plane to Go(ave. R + L divided by distance from Axial plane to Gn
19.	Posterior/anterior vertical ratio (mandibular level)	Distance Co-Go (ave. R+L divided by distance ANS to Gn
	**Miscellaneous**	
20.	Wits appraisal	Distance between A-point and molar coronal Construction Plane (minus) distance B-point to molar coronal Construction Plane
21.	Transverse distance, gnathion, to midsagittal plane (Y-axis asymmetry)	Distance from Gnathion perpendicular to Sagittal plane

### Content-validity (Table 3)

Twelve measures had mean validity scores ≥3.5 in all three domains (No. 1, 2, 3, 8, 9, 12, 13, 15, 16, 17, 18 and 21; range of SOR = 57.1 to 90.3). Three measures had mean scores ≥3.5 in 1–2 domains only (No. 11, 14 and 20; range of SOR = 46.3 to 70). Six measures received validity scores <3.5 in all domains (No. 4, 5, 6, 7, 10, and 19; range of SOR = 22.4 to 50.4) (Table 3).

**Table 3:** Content-validity. Six external experts rated the importance of each proposed morphometric measure (5-point Likert scale, endpoints: 1 = disagree, 5 = Strongly agree) within the three domians:1) description of dentofacial growth deviation (descriptive content), 2) Treatment planning, 3) Long-term changes validity. An average validity score ≥ 3.5 was considered of high content-validity. Additionally, the general importance is illustrated by the general strength of outcome measure (VAS 0–100 mm, endpoints: 0 = not important, 100 = extremely important) and rank of importance (1–21, 1 = most important, 21 = least important).

Reference Number of morphometric measure (No.)	Morphometric measures	Descriptive content validity	Treatment planning validity	Long-term changes validity	Strength of recommended measures Mean (std)	Rank of importance
	**Inter-side diff. in bilateral linear distances**					
1.*	Total posterior mandibular height 1	4.7	4.1	4.7	90.3 (7.7)	1
2.*	Total posterior mandibular height 2 (Cranial level)	4	3.8	3.5	61 (24.3)	10
3.*	Condylar height	3.8	3.5	3.8	62.3 (39.9)	8
4.**	Gonion sagittal distance	2.8	2.5	2.5	36 (31.7)	19
5.**	Transverse width to gonion	2.7	2.5	2.5	22.4 (20.6)	20
6.**	Mandibular basal length	3.3	3.2	3	50.4 (28.2)	16
7.**	Total mandibular length	3	3	2.8	40.2 (29.4)	17
8.*	Maxillary occlusal canting	3.8	3.8	3.8	58 (38.8)	14
9.*	Mandibular occlusal canting	4.3	4.3	4.3	76.9 (14.3)	11
	**Inter-side difference in bilateral angles**					
10.**	Mandibular coronal angle (y-axis asymmetry)	3.2	3	3.2	44.3 (42.2)	18
11.#	Gonion angle	3.8	3.2	3.5	70 (28.2)	7
	**Angles between predefined planes**					
12.*	Mandibular axial angle (z-axis asymmetry)	4.3	4.3	4.5	76.4 (32.6)	3
13.*	Mandibular inclination	3.7	3.7	3.6	78.9 (14.4)	2
14.#	Maxillary sagittal position	3	3	3.8	46.3 (33.4)	15
15.*	Mandibular sagittal position	3.7	3.7	3.7	60.1 (24)	6
16.*	Maxillary occlusal inclination	4	4.7	3.8	80.7 (15.6)	5
17.*	Mandibular occlusal inclination	3.8	4.5	4.2	74 (32.2)	4
	**Anterior/posterior face height ratios**					
18.*	Anterior/posterior lower face height ratio (Cranial level)	4	4	3.8	57.1 (23.5)	12
19.#	Anterior/posterior vertical ratio (mandibular level)	2	2.2	2.8	36.8 (33.2)	21
	**Miscellaneous**					
20.#	Wits appraisal	3.7	3.7	3.3	(25.2)	9
21.*	Transverse distance, gnathion, to midsagittal plane	3.8	4.3	4	77.5 (25)	13

* = score ≥3.5 in all three domains.

** = < 3.5 in all three domains.

# = ≥ in one or two domains.

### Test of reliability (Table 4)

Sixteen measures had intra-rater ICC >0.70, indicating an acceptable level of agreement (No. 1, 2, 3, 7, 8, 11, 12, 13, 14, 15, 16, 17, 18, 19, 20 and 21). Thirteen measures had inter-rater ICC >0.70, indicating an acceptable level of agreement (No. 1, 2, 3, 7, 9, 13, 14, 15, 16, 17, 18, 19 and 20). Convergence of variables with intra-rater and inter-rater ICC >0.70 was seen in 71% (12/17) of the measures that had at least one ICC >0.70 (Table 4).

**Table 4:** Reliability tests. Intra-rater and inter-rater values based on 30 duplicate measurements. An intra-class correlation coefficient of ≥ 0.70 was considered acceptable. Abbreviations: ICC; intra-class correlation coefficients.

Reference Number of morphometric measure (No.)	Morphometric measures	Intra-rater (ICC)	Inter-rater (ICC)	Smallest detectable difference
	**Inter-side diff. in bilateral linear distances**			
1.	Total posterior mandibular height 1	0.86	0.90	+/- 3.3 mm*
2.	Total posterior mandibular height 2 (Cranial level)	0.84	0.73	+/- 3.9 mm*
3.	Condylar height	0.79	0.80	+/- 5.7 mm*
4.	Gonion sagittal distance	0.29	0.17	+/- 7.7 mm*
5.	Transverse width to gonion	0.63	0.67	+/-6.7 mm*
6.	Mandibular basal length	0.57	0.51	+/- 5.9 mm*
7.	Total mandibular length	0.93	0.96	+/- 2.4 mm*
8.	Maxillary occlusal canting	0.82	0.63	+/- 2.9 mm*
9.	Mandibular occlusal canting	0.63	0.72	+/- 2.4 mm*
	**Inter-side difference in bilateral angles**			
10.	Mandibular coronal angle (y-axis asymmetry)	0.65	0.40	+/- 8.9 deg*
11.	Gonion angle	0.71	0.57	+/- 6.5 deg*
	**Angles between predefined planes**			
12.	Mandibular axial angle (z-axis asymmetry)	0.80	0.66	+/- 2.2 deg
13.	Mandibular inclination	0.96	0.96	+/- 2.8 deg
14.	Maxillary sagittal position	0.82	0.89	+/- 3.2 deg
15.	Mandibular sagittal position	0.79	0.94	+/-2.4 deg
16.	Maxillary occlusal inclination	0.73	0.87	+/- 4.2 deg
17.	Mandibular occlusal inclination	0.77	0.84	+/- 5.4 deg
	**Anterior/posterior face height ratios**			
18.	Anterior/posterior lower face height ratio (Cranial level)	0.92	0.91	+/- 0.02 ratio
19.	Anterior/posterior vertical ratio (mandibular level)	0.94	0.93	+/- 0.1 ratio
	**Miscellaneous**			
20.	Wits appraisal	0.72	0.80	+/- 3.1 mm
21.	Transverse distance, gnathion, to midsagittal plane	0.79	0.63	+/- 4.7 mm

*Inter-side difference: the number indicates the smallest detectable difference when the smallest measure is subtracted the larger contralateral measure. The smallest detectable difference quantifies the error of the method and defines the smallest amount of change that can be reliably observed between two consecutive observations.

### Construct validity (Table 5)

The 89 included subjects were divided into four groups: JIA 1 (n = 17, mean age 12.4 ± 2.3 years), JIA 2 (n = 24, mean age 11.3 ± 2.6 years), JIA 3 (n = 29, mean age 12.2 ± 3.4 years), and Control (n = 19, mean age 13.0 ± 2.1 years). The results of construct validity testing are displayed in Table 5.

**Table 5:** Construct validity. ANOVA tests analyzing inter-group differences between controls and three unique JIA groups. Level of significance p = 0.05. Secondary post-ANOVA tests were only performed for outcome variables in which a statistically significant difference was observed in the primary ANOVA test.

Reference Number (No.)	Morphometric measures	0Non-JIA controls (SD)	JIA 1 Normal TMJs (SD)	JIA 2 Unilateral abnormal TMJ (SD)	JIA 3 Bilateral abnormal TMJs (SD)	Anovas	Post-tests
	**Inter-side diff. in bilateral linear distances**						
1.	Total posterior mandibular height 1	-1.0 (2.5)	-3.2 (2.6)	-5.1 (6.2)	-3.9 (3.4)	0.02	0>(JIA1 = JIA2 = JIA3)
2.	Total posterior mandibular height 2 (Cranial level)	0.08 (2.2)	-2.2 (2.3)	-3.3 (5.3)	-2.7 (2.3)	0.01	0>(JIA1 = JIA2 = JIA3)
3.	Condylar height	0.9 (7.2)	-0.3 (5.7)	-0.9 (6.0)	-3.9 (8.8)	0.12	a
4.	Gonion sagittal distance	0.1 (3.5)	0.8 (2.3)	-1.7 (3.5)	1.3 (3.2)	0.01	JIA1>JIA2<JIA3
5.	Transverse width to gonion	0.3 (3.3)	2.1 (3.5)	2.7 (4.3)	2.1 (2.9)	0.17	a
6.	Mandibular basal length	-0.3 (3.0)	0.3 (2.2)	-0.2 (3.6)	0.3 (4.0)	0.88	a
7.	Total mandibular length	1.0 (2.9)	-0.2 (3.3)	0.7 (6.2)	1.4 (4.9)	0.72	a
8.	Maxillary occlusal canting	0.6 (1.5)	-1.2 (1.9)	-1.6 (2.9)	-1.4 (1.9)	0.007	0>(JIA1 = JIA2 = JIA3)
9.	Mandibular occlusal canting	0.1 (1.3)	-1.4 (1.6)	-1.6 (2.7)	-1.6 (1.8)	0.02	0>(JIA1 = JIA2 = JIA3)
	**Inter-side difference in bilateral angles**						
10.	Mandibular coronal angle (y-axis asymmetry)	-0.3 (4.1)	1.0 (2.4)	0.4 (4.8)	1.1 (3.8)	0.63	a
11.	Gonion angle	0.5 (2.8)	-1.2 (3.4)	-1.8 (4.1)	-0.9 (4.0)	0.25	a
	**Angles between predefined planes**						
12.	Mandibular axial angle (z-axis asymmetry)	1.2 (0.8)	1.7 (1.2)	3.8 (2.8)	2.0 (1.5)	0.004	(0 = JIA1) <JIA2>JIA3
13.	Mandibular inclination	28.0 (5.9)	28.3 (4.8)	30.9 (6.8)	35.5 (5.9)	0.0001	(0 = JIA1 = JIA2)<JIA3
14.	Maxillary sagittal position	83.5 (3.3)	83.9 (3.7)	83.3 (4.2)	83.8 (4.5)	0.96	a
15.	Mandibular sagittal position	82.1 (3.3)	82.1 (4.5)	80.2 (4.7)	77.8 (4.9)	0.0032	(0 = JIA1 = JIA2)>JIA3
16.	Maxillary occlusal inclination	16.3 (3.6)	16.8 (4.9)	18.6 (5.3)	20.1 (5.5)	0.04	(0 = JIA1)<JIA3
17.	Mandibular occlusal inclination	11.7 (5.6)	13.0 (5.1)	11.9 (5.6)	17.4 (5.7)	0.0009	(0 = JIA1 = JIA2)<JIA3
	**Anterior/posterior face height ratios**						
18.	Anterior/posterior lower face height ratio (Cranial level)	0.74 (0.04)	0.75 (0.03)	0.74 (0.05)	0.71 (0.04)	0.0008	(0 = JIA1 = JIA2)<JIA3
19.	Anterior/posterior vertical ratio (mandibular level)	1.0 (0.1)	0.99 (0.07)	0.92 (0.09)	0.87 (0.08)	0.0001	(0 = JIA1)>(JIA2 = JIA3)
	**Miscellaneous**						
20.	Wits appraisal	-1.0 (1.9)	-1.4 (3.0)	-0.4 (3.5)	0.7 (2.65)	0.07	a
21.	Transverse distance, gnathion, to midsagittal plane	-0.6 (1.4)	-0.4 (2.9)	-0.01 (4.24)	-0.1 (3.1)	0.94	a

H1 was accepted: larger inter-side differences in posterior mandibular height were observed in the three JIA groups compared to controls (Table 5, No. 1 and 2). However, no significant inter-group differences in condylar height measures were seen (No.3).
H2 was accepted: greater inter-side vertical mandibular was seen in the JIA 2 and JIA 3 groups compared to JIA 1 subjects as illustrated by the inter-group differences in the mandibular axial angle (No. 12).
H3 was partially accepted: Steeper occlusal plane canting (No. 8, 9) was found in JIA groups compared to controls, but not between the 3 JIA groups. As hypothesized, occlusion inclinations were significantly steeper in the JIA 3 group compared to JIA 1 and controls (No.16, 17).
H4 was accepted: Mandibular retrognathia was more significant in the JIA 3 group compared to the other JIA groups and controls (No. 13, 15, 17 and 18).

### Establishment of final recommendations (Tables 6 and 7)

Table 6 depicts the final recommendations within each domain. Seven measures received a “highly recommended” grade within at least one domain (No.1, 8, 9, 12, 13, 17, 18). Of these, 86% (6/7) were highly recommended in all three domains. Nine measures were “moderately recommended” in at least one domain (No. 2, 3, 6. 11, 14–16, 20, 21), and five received a maximum score of “minor recommendation” within one or more domain (No. 4,5,7,10). The mandibular coronal angle was the only measure to receive “not recommended” in terms of therapeutic efficacy validity (No. 10). The highly recommended measures included: inter-side difference in posterior mandibular height, occlusal cant, mandibular asymmetry, mandibular inclination, and anterior/posterior lower facial heights. Table 7 depicts the seven highly recommended measures with a reference to the morphometric growth deviation each measure is intended to assess. The highly recommended measures represent seven unique aspects of abnormal dentofacial growth with no overlap (Table 7).

**Table 6:** Final recommendations. Validity and reliability results were used for the establishment of final recommendation. Each morphometric measure was assigned with a grade of recommendation within each of the domains: highly recommended (+++), moderately recommended (++), somewhat recommended (+), not recommended (-).

Reference Number (No.)	Morphometric measures	Descriptive content validity	Treatment planning validity	Long-term changes validity
	**Inter-side diff. in bilateral linear distances**			
1.	Total posterior mandibular height 1	+++	+++	+++
2.	Total posterior mandibular height 2 (Cranial level)	++	++	++
3.	Condylar height	++	++	++
4.	Gonion sagittal distance	+	+	+
5.	Transverse width to gonion	+	+	+
6.	Mandibular basal length	++	++	+
7.	Total mandibular length	+	+	+
8.	Maxillary occlusal canting	+++	+++	+++
9.	Mandibular occlusal canting	+++	+++	+++
	**Inter-side difference in bilateral angles**			
10.	Mandibular coronal angle (y-axis asymmetry)	+	+	-
11.	Gonion angle	++	+	+
	**Angles between predefined planes**			
12.	Mandibular axial angle (z-axis asymmetry)	+++	+++	++
13.	Mandibular inclination	+++	+++	+++
14.	Maxillary sagittal position	+	+	++
15.	Mandibular sagittal position	++	++	++
16.	Maxillary occlusal inclination	++	++	++
17.	Mandibular occlusal inclination	+++	+++	+++
	**Anterior/posterior face height ratios**			
18.	Anterior/posterior lower face height ratio (Cranial level)	+++	+++	+++
19.	Anterior/posterior vertical ratio (mandibular level)	+	+	+
	**Miscellaneous**			
20.	Wits appraisal	++	+	+
21.	Transverse distance, gnathion, to midsagittal plane	++	++	++

**Table 7:** Highly recommended morphometric measures. Morphometric measures highly recommended for 3D assessment of dentofacial growth deviation in JIA.

Reference Number (No.)	Morphometric measures	Growth deviation assessed
1.	Total posterior mandibular height 1	Inter-side difference in mandibular vertical development
8.	Maxillary occlusal canting	Canting of the maxillary occlusal plane
9.	Mandibular occlusal canting	Canting of the mandibular occlusal plane
12.	Mandibular axial angle (z-axis asymmetry)	Canting of the mandibular lower border with reference to gonion points
13.	Mandibular inclination	Mandibular inclination and assessment of mandibular rotation with reference to mandibular base
17.	Mandibular occlusal inclination	Inclination of mandibular occlusal plane
18.	Anterior/posterior lower face height ratio (cranial level)	Anterior lower face development

## Discussion

Temporomandibular joint involvement is a frequent finding in JIA [2, 3] and often impacts dentofacial growth. This belies the importance of establishing standardized recommendations for the evaluation of growth deviation in this population. In this study, we evaluated 21 morphometric measures using an established five-step method. To our knowledge, this is the first study to evaluate such measures in the JIA population.

Seven measures received a high recommendation in all three domains and should therefore be considered of great importance for the study of dentofacial growth in JIA. These include: inter-side difference in posterior mandibular height, occlusal cant, mandibular asymmetry, mandibular inclination, and anterior/posterior lower facial heights. Future work will include establishing an index to assess the severity of dentofacial growth deviation in JIA for these seven highly recommended measures.

Despite many prior publications regarding 3D dentofacial imaging landmark identification and reproducibility, a systematic review from Pittayapat et al. concluded that additional standardization is necessary [9]. Prior studies fail to relate facial asymmetry with the error of the method. In the present study, we calculated the smallest detectable difference for each measure in order to define the minimum discernable change between two observations [29, 30]. Surprisingly, we found high values for the smallest detectable differences of many previously published measures (Table 4). For example, 2D analyses concluded that condylar height was one of the most important measurements in the assessment of dentofacial growth deviation [25, 32], but the smallest detectable difference for this measure in our study was found to be 5.7 mm. We hypothesize that this is due to large variation in landmark identification of the condylion point due either to condylar deformity from JIA or natural variability in condylar shape [33].

Construct validity was achieved by acceptance of the hypothesis that dentofacial growth deviation was more pronounced in the JIA groups compared to controls. However, contrary to our expectations, we did not observe inter-group differences between the three JIA groups in asymmetry-based measures like total posterior height 1 and 2 (No.1,2), and maxillary and mandibular occlusal canting (No.8,9). This could be explained by the classification of the JIA groups based on radiographic appearance; contemporary theory explains the development of dentofacial growth deviation in JIA as a consequence of condylar growth disturbance rather than condylar damage [4, 17, 34–36]. Therefore, the radiographic appearance of the condyle may be normal even when growth at that condyle has been impaired. This is in agreement with findings of Twilt et al., reporting dysmorphic mandibular development in patients with JIA without detectable condylar abnormalities on orthopantomograms [35]. It was also surprising that no differences were observed between the JIA groups and controls for condylar height (No.3), mandibular basal length (No.6), and Wits appraisal (No.20). This could also be due to the variation in landmark identification for these points, which is illustrated in the large values for smallest detectable differences for these variables. These findings reveal a pitfall of morphometric facial analysis which should be considered in future studies: statistical significance may not indicate clinical relevance if error of the method is not considered.

This study has several limitations. Inter-side differences were used to express the degree of asymmetry [11, 14, 37], but were not related to normal variation associated with age. These results may therefore be misleading as, for example, a total posterior mandibular height inter-side difference of 4 mm may be a severe sign of asymmetry in an 8-year-old, but less significant in a 17-year-old patient. An alternative approach would have been to express asymmetry as a ratio, but this would have limited clinical applicability. Additionally, we defined the “asymmetry-side”, which serves as the basis for other calculations, as the side with the smallest total posterior mandibular height. However, this side may not have had the smallest linear distance or angle for all measurements. Nonetheless, this allowed consistent comparisons between sides and facilitated a complete characterization of facial asymmetry. Color-coded overlay mapping is an alternative technique for 3D visualization of facial asymmetry with promising clinical and research applicability [38, 39].

As demonstrated by Liukkonen et al., mandibular asymmetry is common, even in a non-JIA population. This asymmetry is most often clinically irrelevant and frequently improves with age [40]. Although we demonstrated an inter-group difference in dentofacial symmetry between JIA2/JIA3 and the control subjects, this exemplifies a general limitation to this study: there is a lack of standardized, consensus-based recommendations for radiographic examination in the JIA population.

In our current practice, CBCT examinations are obtained when there is clinical suspicion for a dentofacial abnormality, such as a TMJ hard-tissue anomaly, a progressive dentofacial asymmetry, or for treatment decision making. Increasingly, CBCT is becoming routine in orthodontic and orthognathic surgical treatment planning and quality assessment. This expansion is fueled by the increasing availability of CBCT machines and the reduction in radiation dose required to obtain an adequate image, which is considerably lower than exposure from medical CT [41]. Recent publication by Markic et al. have compared alternative techniques for 3D visualization of facial asymmetry (orthopantomograms, CBCT, CT and magnetic resonance imaging (MRI)) and found equal abilities of the methods to assess inter-side difference in mandibular development [42]. Future research is needed to validate, if the morphometric measures recommended in the present study can be applied to MRI examination, allowing the elimination of ionizing radiation. and potentially the combination of soft and hard-tissue assessment into a single study.

In conclusion, we have identified and validated a series of morphometric measures for the assessment of dentofacial growth deviation in patients with JIA. Seven measures received a “high recommendation” score. Those measures were associated with posterior mandibular height, occlusal cant, mandibular asymmetry, mandibular inclination, and anterior/posterior lower facial height. These measurements will facilitate standardization of radiographic analysis in this population. This work offers important insight to the dentofacial consequences of TMJ arthritis in growing individuals and provides a framework for future research.
